# The relationship of the medial roof and the posterior wall of the maxillary sinus to the sphenoid sinus: a radiologic study^☆^

**DOI:** 10.1016/j.bjorl.2016.04.007

**Published:** 2016-05-09

**Authors:** Seung Ju Lee

**Affiliations:** Eulji Hospital, Eulji University, College of Medicine, Department of Otorhinolaryngology, Head and Neck Surgery, Seoul, South Korea

**Keywords:** Computed tomography, Medial maxillary sinus roof, Posterior wall of maxillary sinus, Sphenoid sinus, Tomografia computadorizada, Teto medial do seio maxilar, Parede posterior do seio maxilar, Seio esfenoide

## Abstract

**Introduction:**

The medial maxillary sinus roof is a ridge formed by the superior margin of the maxillary sinus antrostomy. The posterior wall of the maxillary sinus is always included in operative fields.

**Objective:**

To perform a radiologic study assessing the utility of the medial maxillary sinus roof and the posterior wall of the maxillary sinus as fixed landmarks for providing a safe route of entry into the sphenoid sinus.

**Methods:**

We reviewed 115 consecutive paranasal sinus Computed Tomographic scans (230 sides) of Korean adult patients performed from January 2014 to December 2014. Using the nasal floor as a reference point, the vertical distances to the highest point of the medial maxillary sinus roof, the sphenoid ostium and anterior sphenoid roof and floor were measured. Then the vertical distances from the highest point of the medial maxillary sinus roof to the sphenoid ostium and anterior sphenoid roof and floor were calculated. The coronal distance from the posterior wall of the maxillary sinus to the sphenoid ostium was determined.

**Results:**

The average height of the highest point of the medial maxillary sinus roof relative to the nasal floor was measured to be 33.83 ± 3.40 mm. The average vertical distance from the highest point of the medial maxillary sinus roof to the sphenoid ostium and anterior sphenoid roof and floor was 1.79 ± 3.09 mm, 12.02 ± 2.93 mm, and 6.18 ± 2.88 mm respectively. The average coronal distance from the posterior wall of the maxillary sinus to the sphenoid ostium was 0.78 mm. The sphenoid ostium was behind the coronal plane of the posterior wall of the maxillary sinus most frequently in 103 sides (44.4%). It was in the same coronal plane in 68 sides (29.3%) and in front of the plane in 61 sides (26.3%).

**Conclusions:**

The medial maxillary sinus roof and the posterior wall of the maxillary sinus can be used as a reliable landmark to localize and to enable a safe entry into the sphenoid sinus.

## Resumo

**Introdução:**

O teto medial do seio maxilar é uma crista formada pela margem superior da antrostomia do seio maxilar. A parede posterior do seio maxilar é sempre incluída em campos cirúrgicos.

**Objetivo:**

Realizar estudo radiológico avaliando a utilidade do teto medial do seio maxilar e da parede posterior do seio maxilar como referências anatômicas fixas para fornecer uma via segura de abordagem ao seio esfenoidal.

**Método:**

Foram analisados 115 exames de tomografia computadorizada consecutivos dos seios paranasais (230 lados) de pacientes adultos coreanos realizados de janeiro de 2014 a dezembro de 2014. Usando o assoalho nasal como ponto de referência, as distâncias verticais entre o teto medial do seio maxilar e o óstio esfenoidal e entre o teto e o assoalho esfenoidal anterior foram medidas. Em seguida, as distâncias verticais do ponto mais alto do teto medial do seio maxilar e o óstio esfenoidal e entre o teto e ao assoalho esfenoidal anterior foram medidas. A distância coronal da parede posterior do seio maxilar ao óstio esfenoidal foi determinada.

**Resultados:**

A altura média do ponto mais alto do teto medial do seio maxilar em relação ao assoalho nasal foi medida como sendo 33,83 ± 3,40 mm. A distância vertical média do ponto mais alto do teto medial do seio maxilar até o óstio esfenoidal e do teto ao assoalho esfenoidal anterior foi de 1,79 ± 3,09 mm, de 12,02 ± 2,93 mm e 6,18 ± 2,88 mm, respectivamente. A distância coronal média da parede posterior do seio maxilar ao óstio esfenoidal foi de 0,78 mm. O óstio esfenoidal estava por trás do plano coronal da parede posterior do seio maxilar com mais frequência em 103 lados (44,4%). O mesmo se encontrava no plano coronal em 68 lados (29,3%) e na frente do plano em 61 lados (26,3%).

**Conclusões:**

O teto medial do seio maxilar e a parede posterior do seio maxilar podem ser usados como pontos de referência confiáveis para localizar e possibilitar uma abordagem segura ao seio esfenoidal.

## Introduction

For safe and effective Endoscopic Sinus Surgery (ESS), preoperative evaluation of Computed Tomographic (CT) scans are required to identify the extent of disease and critical anatomic landmarks. However, during real operations, landmarks on CT scans often have significant anatomic variations or are obscured by blood, polyps or other inflammatory or postsurgical changes. Accordingly, other more unchanging and fixed anatomic landmarks are needed. The ideal anatomic landmarks must be consistent, easy to find even in the distorted nasal cavities, and provide the surgeon with a sense of direction as one proceeds posteriorly.

The medial maxillary sinus roof (MMSR) is a ridge formed by the superior margin of the maxillary sinus antrostomy and represents the level of the medial orbital floor.^1^, ^2^, ^3^ It is bordered by the inferior edge of the lamina papyracea, and has been an useful landmark for finding the lamina papyracea.^4^ It is easily identifiable during ESS in spite of previous surgery or severe inflammatory disease of nasal cavity and paranasal sinuses. Recently, this ridge has been regarded as an important intraoperative landmark in locating the sphenoid sinus.^1^, ^2^, ^3^

Casiano measured the vertical distance from the posterior medial orbital floor to the sphenoid sinus floor and the sphenoid sinus height on human cadaver heads. He concluded that the sphenoid sinus will be entered consistently at the location of the sphenoid ostium at the level of the posterior medial orbital floor.^1^ In Harvey et al.’s study, the maxillary sinus roof could be used as a robust landmark to enable a safe entry to the sphenoid sinus when normal structures are not available.^2^ In Lee et al.’s study, the MMSR was a reliable preoperative reference point for guiding safe surgical entry into the sphenoid sinus.^3^ The conclusions of Harvey et al. and Lee et al. were based on the CT study, but few objective data are available at this moment.

The posterior wall of the maxillary sinus which is always included in the operative fields is also easy to find and use. Thus, it may also serve as a reliable landmark in locating the anterior wall of the sphenoid sinus. In the previous study of Casiano, the posterior wall of the maxillary sinus is several millimeters in front of the approximate level of the anterior sphenoid wall in the coronal plane. He suggested that the posterior wall of the maxillary sinus as seen through the antrostomy, demarcate the approximate level, in the coronal plane, of the anterior wall of the sphenoid sinus.^1^ But, to the best of our knowledge, there has been no detailed study concerning the coronal distance from the posterior wall of maxillary sinus to the sphenoid ostium.

The aim of this study was to perform radiologic study assessing the utility of the MMSR and the posterior wall of the maxillary sinus as fixed landmarks for providing a safe route of entry into the sphenoid sinus.

## Methods

We retrospectively reviewed 115 consecutive paranasal sinus triplanar CT scans of Korean adult patients performed from January 2014 to December 2014, giving a total of 230 sides for analysis. Each patient's information such as age, gender, and the purpose of the scan has been collected. These patients underwent CT scanning for the assessment of various nasal symptoms such as nasal obstruction, rhinorrhea, posterior nasal dripping, anosmia, facial pain, headache, etc. Patients requiring ESS with CT findings of chronic paranasal sinusitis were excluded. Scans with haziness of posterior ethmoid or sphenoid sinuses were also excluded. Scans were excluded if there was alteration of the posterior ethmoid or sphenoid sinus skull base either from previous surgery or from other noninflammatory conditions. In addition, scans were excluded if the ostium of the sphenoid sinus was not identified on the sagittal CT scan. The study protocol for a retrospective CT review for research purposes was exempt from the Institutional Review Board review.

CT scans of the nasal cavities and paranasal sinuses were performed with contiguous axial cuts of 2.5 mm thickness (120 kVp, 220mAs, FOV 180 mm × 180 mm) on GE Discovery 750HD CT scanners (GE Medical Systems, Milwaukee, WI, USA). The CT data were then reconstructed into coronal and sagittal images of 2 mm thickness by GE workstation Adw 4.5 software (GE, USA). Measurements were done as follows:

1. Maximum height of the MMSR relative to the nasal floor (MS-NF) (Fig. 1A). The highest MMSR (hMMSR) was found by scrolling through the coronal images. The slice in which the MMSR was highest relative to the nasal floor within any area of the maxillary sinus was identified. The vertical distance from the nasal floor to the hMMSR was measured.

**Figure 1:**
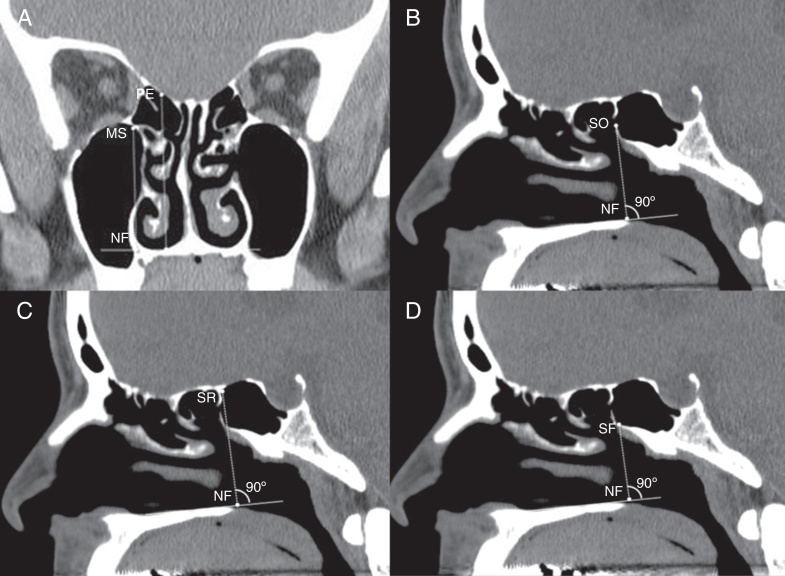
(A) Coronal CT image demonstrating the vertical distance from the nasal floor (NF) to the highest medial maxillary sinus roof (MS) and posterior ethmoid skull base (PE). (B) Sagittal CT image demonstrating the vertical distance from the nasal floor (NF) to the sphenoid ostium (SO). (C) Sagittal CT image demonstrating the vertical distance from the nasal floor (NF) to the anterior sphenoid sinus roof (SR). (D) Sagittal CT image demonstrating the vertical distance from the nasal floor (NF) to the anterior sphenoid sinus floor (SF).
2. Vertical distance from the inferior margin of the sphenoid ostium to the hMMSR (SO-MS). Bony opening at the sphenoid face was identified both on axial and sagittal CT images. The sagittal slice centered on the sphenoid ostium was used to measure the vertical distance from the nasal floor to the sphenoid ostium (SO-NF) (Fig. 1B). SO-MS was calculated by subtracting MS-NF from SO-NF. A sphenoid ostium inferior to the hMMSR was given a negative value.
3. Vertical distance from the anterior sphenoid sinus roof to the hMMSR (SR-MS). The sinus roof was identified on a same sagittal CT slice centered on the sphenoid ostium. The vertical distance from the nasal floor to sphenoid sinus roof was measured (SR-NF) (Fig. 1C). If an Onodi cell was found, the vertical distance was measured from the nasal floor to the skull base at the coronal plane of the anterior sphenoid face. SR-MS was calculated by subtracting MS-NF from SR-NF.
4. Vertical distance from the hMMSR to the anterior sphenoid sinus floor (MS-SF). The sinus floor was identified on a same sagittal CT slice centered on the sphenoid ostium. The vertical distance from the nasal floor to sphenoid sinus floor was measured (SF-NF) (Fig. 1D). MS-SF was calculated by subtracting SF-NF from MS-NF.
5. The coronal distance from the posterior wall of the maxillary sinus to the sphenoid ostium. Triplanar display of CT data in axial, sagittal, and coronal planes was simultaneously viewed and analyzed. The sphenoid ostium in the axial plane was identified. The most posterior point of the posterior wall of the maxillary sinus was identified by scrolling through the sagittal images. It was usually located at the medial part of maxillary sinus. Using the Picture Archiving and Communication System (PACS) cross-referencing tool, the relationship between the most posterior point of the posterior wall of the maxillary sinus in the sagittal plane and the sphenoid ostium in the axial plane was determined. The sphenoid ostium was behind (Fig. 2A) or in fronts of (Fig. 2B) the coronal plane of the most posterior point of the posterior wall of the maxillary sinus. Or it was in the same plane (Fig. 2C). The distance between the two structures was determined by the number of the intervening 2 mm-thick coronal slices between them. A sphenoid ostium anterior to the coronal plane of the most posterior point of the posterior wall of the maxillary sinus was given a negative value.

**Figure 2:**
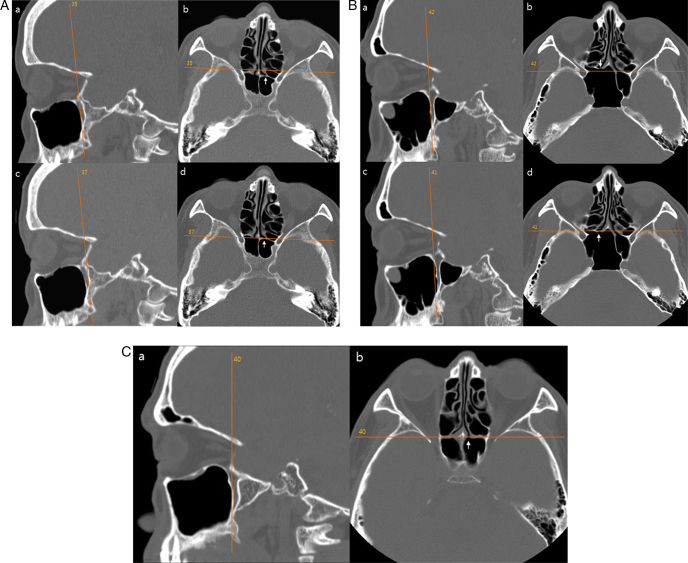
(A) The left sphenoid ostium (arrows in b and d) is 2 slices behind the coronal plane of the posterior wall of the left maxillary sinus (a). (B) The right sphenoid ostium (arrows in b and d) is one slice in front of the coronal plane of the posterior wall of the right maxillary sinus (a). (C) The left sphenoid ostium (b) is in the same coronal plane of the posterior wall of the left maxillary sinus (a).

Measurements were made for both the right and left sides of each patient. Paired *t* tests were used to indicate whether mean differences existed between right and left sides. Statistical analysis was done using SPSS version 15 (SPSS Inc., Chicago, IL). Statistical significance was defined as *p* < 0.05.

## Results

Measurements were taken from 115 CT scans, giving a total of 230 sides for analysis. The study group consisted of 53 (46.1%) females and 62 (53.9%) males. The mean patient age was 40.4 years (range, 18–81 years). *T*-test did not demonstrate any significant difference between the right and left sides in any distance measured in this study (*p* > 0.05). The mean, standard deviation and range of the measurements are seen in Table 1.

**Table 1:** Summary of vertical distances in our study.

Measurement	Mean (mm) ± SD	Range (mm)
hMMSR – nasal floor	33.83 ± 3.40	26.37 to 43.98
hMMSR – sphenoid ostium	1.79 ± 3.09	−5.60 to 10.84
hMMSR – sphenoid roof	12.02 ± 2.93	4.22 to 18.29
hMMSR – sphenoid floor	6.18 ± 2.88	1.06 to 12.85

hMMSR, highest medial maxillary sinus roof; SD, standard deviation.

The average height of the highest MMSR relative to the nasal floor was measured to be 33.83 ± 3.40 mm, ranging from 26.37 to 43.98 mm. The average vertical distance from the sphenoid ostium to the highest MMSR was 1.79 ± 3.09 mm, ranging from −5.60 to 10.84 mm. The average vertical distance from the anterior sphenoid sinus roof to the highest MMSR was 12.02 ± 2.93 mm, ranging from 4.22 to 18.29 mm. The average vertical distance from the highest MMSR to the anterior sphenoid sinus floor was 6.18 ± 2.88 mm, ranging from 1.06 to 12.85 mm.

**Figure 3:**
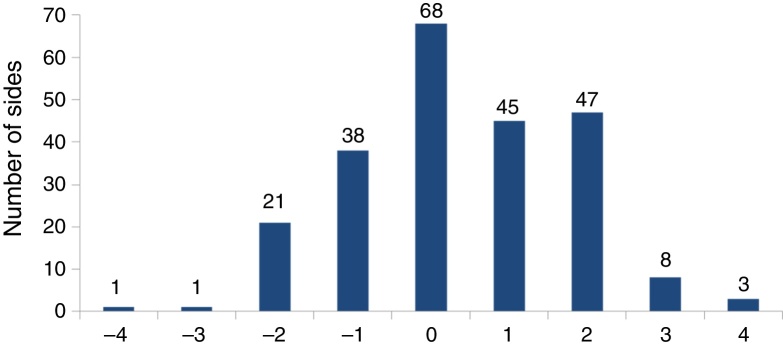
The distribution of the number of the 2 mm-thick slices between the coronal plane of the posterior wall of the maxillary sinus and the sphenoid ostium. A sphenoid ostium anterior to the coronal plane of the most posterior point of the posterior wall of the maxillary sinus was given a negative value.

The distribution of the number of the slices between the coronal plane of the posterior wall of the maxillary sinus and the sphenoid ostium is displayed in Fig. 3. The sphenoid ostium was behind the coronal plane of the posterior wall of the maxillary sinus most frequently in 103 sides (44.4%). It was in the same coronal plane in 68 sides (29.3%) and in front of the plane in 61 sides (26.3%). On average, 0.39 coronal slice was present between the two structures with a standard deviation of 1.42 slices. As the CT slice thickness in our study was 2 mm, the average distance between the two structures was 0.78 mm.

## Discussion

In this study, the mean of the maximum height of the MMSR was measured to be 33.83 mm, very similar to 33.45 mm in Lee et al.’s study and 33.9 mm in Harvey et al.’s study.^2^, ^3^ We think that the highest MMSR can be easily identified endoscopically at this height relative to the nasal floor after maxillary antrostomy are performed.

To enter the sphenoid sinus safely, its size, shape, and relationship to the optic nerve and carotid artery should be determined by reviewing triplanar CT scans, preoperatively. The sphenoid sinus can be safely entered inferiorly and medially, above the arch of the posterior nasal choana and close to the posterior aspect of the septum.^4^, ^5^ The superior turbinate served as a key landmark.^6^, ^7^, ^8^ However, when the superior turbinate is not available, previously resected or replaced by pathology, superior dissection will potentially damage the skull base. This may be especially important when the ostium cannot be visualized either due to bleeding, polyps, and inflammatory disease or if technologies such as image guidance navigation are not available. In this situation, other landmarks are needed.

In Casiano's study with cadaveric model, the medial orbital floor was noted as 39% of the sphenoid height. He suggested that at the level of the posterior medial orbital floor, the sphenoid sinus will be entered consistently in its inferior to middle third, which also corresponds to the location of the sphenoid ostium in most cases.^1^ Recently, it has become possible to evaluate CT images three-dimensionally with the advancement of multiplanar reconstruction technique. Sagittal CT images can help us promote the safety of ESS. In the first radiologic study by Lee et al., relative to the maxillary sinus roof, the sphenoid ostium was identified at a vertical height 2.76 mm superior to the highest MMSR.^3^ In this three-dimensional radiologic study, the sphenoid ostium was identified at a vertical height 1.79 mm superior to the highest MMSR, ranging from −5.60 to 10.84 mm. Our result is 0.97 mm shorter than that in the previous report. Thus, there is quite a small difference between our result and that of the previous report.

The mean vertical distance from the highest MMSR to the anterior sphenoid roof at the sagittal slice in which the sphenoid ostium was identified in our study (12.02 mm) was nearly identical to that in the previous study (12 mm).^3^ Harvey et al. measured at the sagittal plane centered on the superior turbinate, and the distance was 11.77 mm on average.^2^ The anterior sphenoid sinus floor is approximately 6 mm inferior to the maxillary sinus roof in Lee et al.’s study and 6.18 mm in the current study.^3^

Taken together, the findings of our study corroborate earlier observations. To simplify the data in this and previous studies, the ostium of the sphenoid sinus and the sphenoid sinus roof are about 2–3 mm and 11–12 mm superior to the highest MMSR and the sphenoid sinus floor is about 6 mm inferior to the highest MMSR. We think that the sphenoid ostium can be found easily at the height of the highest MMSR and this relationship is helpful especially when the sphenoid sinus is entered by transethmoidal approach. Therefore, the MMSR can be a good landmark for entry to the sphenoid sinus while it is kept in view on the monitor screen and referred to throughout the surgery.

In our measurements, the minimum distance between the MMSR and sphenoid sinus roof was 4.22 mm. The MMSR may be always below the sphenoid skull base. Thus, the MMSR provides a reasonable margin of safety from the skull base and the sphenoid sinus can be entered safely without damage to the skull base at the height of the highest MMSR. Lastly, attempting to enter the sphenoid too inferiorly relative to the MMSR can be difficult due to thicker bone of sphenoclival junction.

Casiano performed direct measurements after sectioning the cadaver heads sagittally in the midline and endoscopic measurements using straight and curved suction tip.^1^ The anterior face of the sphenoid and the posterior wall of the maxillary sinus was found to be approximately 70 mm from the base of the columella.

In all specimens included in Casiano's study, the anterior face of sphenoid (at the medial orbital floor level) was approximately 2–4 mm more posterior, in the coronal plane, than the posterior wall of the maxillary sinus. But, in this three-dimensional radiologic study, the average coronal distance between the two structures was 0.78 mm, much shorter than the previous result. One of the reasons for this difference might be the difference in the measurement method used. Remarkably, though the sphenoid ostium was behind the coronal plane of the posterior wall of the maxillary sinus most frequently (44.4%), our study showed that it may be in the same coronal plane or in front of the coronal plane of the posterior wall of the maxillary sinus. To the best of our knowledge, this is the first report measuring the coronal distance between the posterior wall of the maxillary sinus and the sphenoid ostium. We think that this anatomic relationship along with the MMSR will be of great assistance to ESS surgeons. The ostium of sphenoid sinus can be much easily identified just above the height of the MMSR and just behind the coronal plane of the posterior wall of maxillary sinus.

Above mentioned mean values in this and the previous publications are not absolute. As shown in Table 1 and Fig. 3, the vertical and coronal distances of each patient can vary greatly with relatively wide range. However, the differences in measurements were within a few millimeters on average, suggesting that these differences may not affect the overall clinical use when performing ESS. The individual values for each patient which show large variation according to the age, sex, and the degree of the maxillary sinus pneumatization should be kept in mind while performing sphenoidotomy using the MMSR and the posterior wall of the maxillary sinus as fixed anatomical landmarks.

## Conclusions

The MMSR and the posterior wall of the maxillary sinus can be used as a reliable landmark to localize and to enable a safe entry into the sphenoid sinus.

## Conflicts of interest

The author declares no conflicts of interest.
